# Extramedullary Acute Myeloid Leukemia: Leukemic Pleural Effusion, Case Report and Review of the Literature

**DOI:** 10.3389/fonc.2014.00130

**Published:** 2014-06-02

**Authors:** Naveen Pemmaraju, Elaine Chang, Naval Daver, Keyur Patel, Jeffrey Jorgensen, Bradley Sabloff, Srdan Verstovsek, Gautam Borthakur

**Affiliations:** ^1^Department of Leukemia, The University of Texas MD Anderson Cancer Center, Houston, TX, USA; ^2^Department of Pathology, The University of Texas MD Anderson Cancer Center, Houston, TX, USA; ^3^Department of Radiology, The University of Texas MD Anderson Cancer Center, Houston, TX, USA

**Keywords:** extramedullary AML, acute myeloid leukemia, malignant pleural effusion

## Abstract

**Objective and Importance**: Malignant pleural effusions occur in the setting of both solid and hematologic malignancies. Pleural effusion caused by leukemic infiltration is an unusual extramedullary manifestation of acute myeloid leukemia (AML) with fewer than 20 cases reported (1–11). We report a case of pericardial and pleural effusions in a patient with AML and review the literature.

**Clinical Presentation**: In this case, a 55-year-old man with previous history of myeloproliferative neoplasm experienced transformation AML, heralded by appearance of leukemic pleural effusions. The patient was identified to have leukemic pleural effusion based on the extended cytogenetic analysis of the pleural fluid, as morphologic analysis alone was insufficient.

**Intervention:** The patient was treated with hypomethylator-based and intensive chemotherapy strategies, both of which maintained resolution of the effusions in the remission setting.

**Conclusion:** Due to the rarity of diagnosis of leukemic pleural effusions, both cytogenetic and fluorescence *in situ* hybridization testing are recommended. Furthermore, systemic chemotherapy directed at the AML can lead to complete resolution of leukemic pleural effusions.

## Objective and Importance

Pleural effusion caused by leukemic infiltration is an unusual extramedullary manifestation of acute myeloid leukemia (AML), but may be more common than previously thought. Fewer than 20 cases have been reported (1–11). We report a case of pericardial and pleural effusions in a patient with AML and review the literature.

## Case Report: Clinical Presentation

A 55-year-old man with polycythemia vera (PV) had pancytopenia, moderate pericardial effusion, and bilateral pleural effusions. He had an 8-year history of PV treated with hydroxyurea and anagrelide. He was in his usual state of health until 6 weeks prior to presentation to our institution when he went to an outside hospital for a cholecystectomy.

In the weeks following his surgery, he developed nausea and vomiting, and was found to have a duodenal ulcer and esophageal erosions. During treatment for these complications, he was noted to have a moderate-sized pericardial effusion and pancytopenia. A bone marrow biopsy showed progression of PV to AML.

He was referred to our institution with shortness of breath, chest pain, and pancytopenia. Bone marrow biopsy at our institution confirmed the diagnosis of AML with complex cytogenetics including trisomy 8, trisomy 9, trisomy 21, and molecular analysis positive for *JAK2 V617F* mutation.

**Figure 1:**
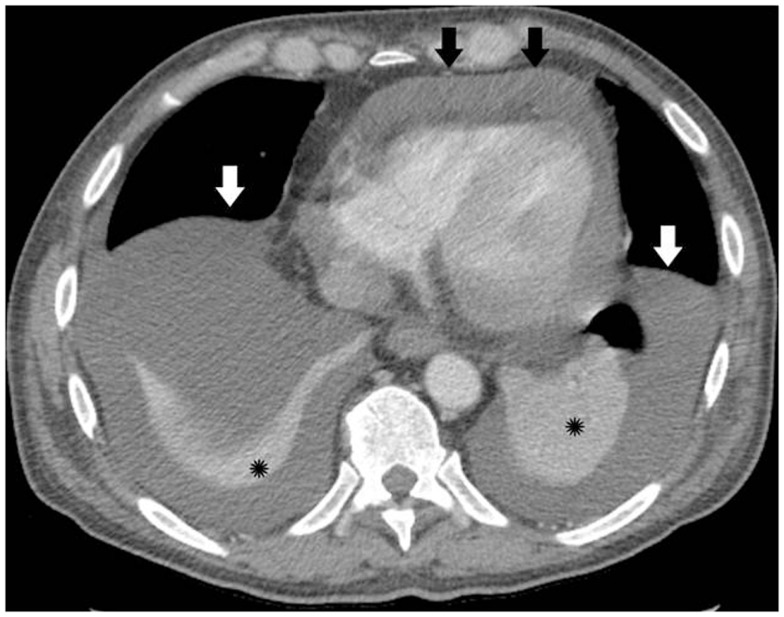
**Pre-treatment computed tomography (CT) image with intravenous (IV) contrast, demonstrating bilateral pleural effusions (white arrows) and moderate-sized pericardial effusion (black arrows), prior to thoracentesis, pericardiocentesis, or chemotherapy**. Black asterisk = atelectatic lung.

The patient had pericardial and exudative pleural effusions (Figure 1) causing increased oxygen requirement. He underwent thoracentesis and pericardiocentesis. Work-up for infectious, rheumatologic, and cardiac causes of the effusions was negative. Cytology of both effusions revealed reactive mesothelial cells in a background of acute inflammation, but no malignant cells were identified. Flow cytometry was done, and the cells in both the pleural and pericardial fluid were predominantly (70%) of a phenotype consistent with basophils. Repeat thoracentesis with cytogenetic analysis was performed. Fluorescent *in situ* hybridization was significant for trisomy 8, trisomy 9, and trisomy 21 in the pleural effusion. These cytogenetic abnormalities were identical to the those detected on the presenting bone marrow, and were found in the majority of the cells analyzed, thereby confirming the presence of leukemic pleural effusions.

## Intervention

**Figure 2:**
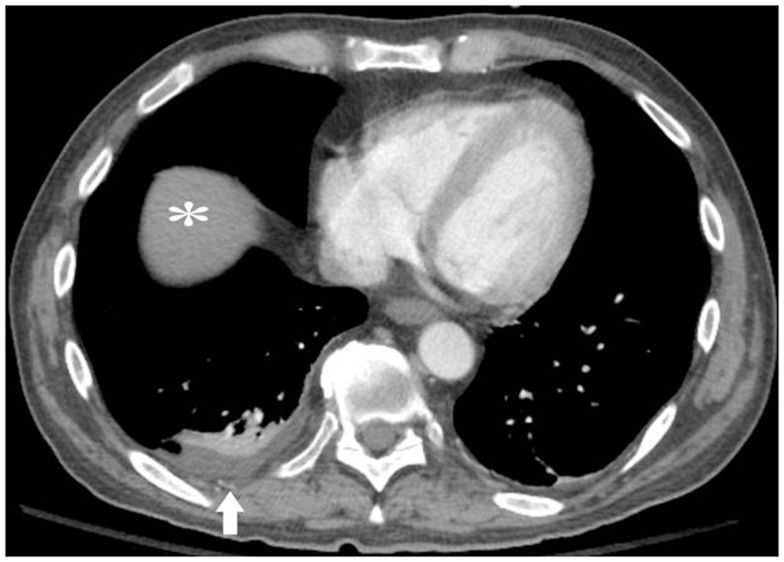
**Post-treatment CT image after two cycles of fludarabine and cytarabine and four cycles of decitabine alone**. White arrow = residual small right pleural effusion. No left pleural effusion or pericardial effusion. White asterisk = liver.

The patient was initially treated with decitabine for 5 days. He had dramatic improvement in performance status during the hospital stay and then also went on to receive twice-daily fludarabine and cytarabine (BIDFA) for an additional 4-day first cycle. He responded with an approximately 50% reduction of blasts and was given a second cycle of therapy with BIDFA for 4 days. Complete remission was achieved after the second cycle, but he developed neutropenic fevers, sepsis, and bacterial endocarditis, with decreased performance status to 2. In the setting of continued complete remission but with decreased performance status and co-morbidities, he was switched to single agent decitabine, which he tolerated well for four cycles. His performance status improved back to 1, and imaging continued to demonstrate near total resolution of effusions in the absence of requiring any further thoracenteses (Figure 2). He was being considered for stem cell transplantation. Unfortunately, the AML ultimately relapsed prior to stem cell transplant, and the patient died 11 months after his transformation to AML in the setting of multi-organ failure and diffused alveolar hemorrhage, although without effusions.

## Discussion

Clinically significant pleural effusions, regardless of etiology, are rarely encountered in patients with AML. A retrospective review of all patients with acute leukemia, myelodysplastic syndrome (MDS), or myeloproliferative neoplasm (MPN) who underwent pleural procedures at our institution from 1997 to 2007 found that 2% (111 patients out of 6,442) required thoracentesis, chest tube insertion, or pleural catheter placement. Sixty-nine of the patients had AML; among these, 39 pleural effusions were infectious and 25 were leukemic, determined by positive cytology or flow cytometry (12). However, the incidence of leukemic pleural effusions may be increasing as a result of longer survival with improved chemotherapies (8). In their 10 year review, Faiz et al. noted that the role of infection was similar to that reported in previous autopsy series, albeit malignant infiltrative effusions were more frequent than in earlier series (12). Our review of the literature yielded 13 individual case reports, summarized in Table 1.

**Table 1:** **Summary of case reports of AML pleural effusion**.

Author (Ref.)	Sex	Age (year)	Leukemia status at leukemic effusion diagnosis	Treatment after diagnosis of leukemic effusion	Overall outcome
Raynolds (1)	M	26	AML	Supportive care	Death 5 months after diagnosis
Ohe et al. (2)	M	51	CD7 + AML	Induction araC + daunorubicin, then autologous HSCT	Complete remission for at least 8 months
Park et al. (3)	M	41	AML recurrence (31 months after) HSCT, in BM remission	Not reported	Not reported
Khan et al. (4)	F	71	AML M5 (acute monocytic leukemia)	Induction araC + daunorubicin	Death 22 days after initiation of induction chemotherapy
Farray et al. (5)	F	45	Acute megakaryoblastic leukemia (M7)	Not reported	Not reported
Fatih (6)	M	50	AML M1	3 + 7 induction idarubicin + cytarabine	Effusion resolved, but leukemia was refractory; death 3 months after discharge from clinic
Huang (7)	F	56	CMMoL with transformation to AML	3 + 7 induction idarubicin + cytarabine	Respiratory failure; death on hospital day 64
Stoll (8)	M	54	AML–MDS	None, ineligible due to renal function	Home hospice; death 1 week after discharge
Ou et al. (9)	M	53	AML with recurrent genetic abnormalities	3 + 7 induction idarubicin + cytarabine	Complete remission with first induction, but effusions did not resolve. After re-induction with high-dose cytarabine, effusions resolved. Later underwent HSCT, remained disease-free
Chang (10)	F	74	AML	Induction cytarabine	Effusions resolved; complete remission for at least 11 months
Chang (10)	M	75	CMMoL with transformation to AML	None, ineligible due to poor performance status	Death 1 month after diagnosis of pleural myeloid sarcoma
Chang (10)	M	74	Refractory AML	Supportive care	Death 1 month after confirmed leukemic pleural effusion
Agrawal (11)	M	45	AML M2	Induction cytarabine	Death 1 week later

*HSCT, hematopoietic stem cell transplant; BM, bone marrow; araC, cytarabine; CMMoL, chronic myelomonocytic leukemia*.

AML is not a common cause of pleural effusions. A 14-year study of 5,888 pleural fluid specimens by Johnston et al. found that 10% of the effusions were malignant, of which 15% were attributable to “leukemia” or “lymphoma,” but more specific histopathologic diagnosis were not available (13). A 2-year retrospective analysis of pleural fluid cytological specimens in India found that of 898 samples, 164 (18%) were malignant, the majority of hematologic malignancies were Non-Hodgkin’s lymphoma, and none were AML (14). Similarly, a more recent review of 4,684 consecutive pleural fluid specimens received in Turkey found only 1 positive for AML (15).

Other sites of extramedullary leukemia (EML) have been associated with FAB M4-M5 subtypes, CD56 (+) blasts, cytogenetic abnormalities including t(8:21), inversion 16, infant leukemia, 11q abnormalities, cellular immune dysfunction, and allogeneic stem cell transplantation. More studies are needed to demonstrate statistically significant relationships with these variables in patients with AML pleural effusions. In their review of AML, ALL, and MDS/MPN collectively, Faiz et al. found that the presence of pleural effusions did not influence survival but did portend more aggressive disease if the hematologic malignancy was diagnosed within 6 months (12).

All the cases described herein occurred concomitantly with or followed the onset of systemic disease, although in some circumstances EML may be discovered prior to the medullary diagnosis. Still, the timing of the development of a pleural effusion varied among these cases, from initial presenting symptom to a component of progression in patients with refractory disease or as a harbinger of relapse in patients with marrow remission. Additionally, as noted before, leukemic pleural effusions can occur as an isolated finding or in association with parenchymal lung disease (16).

This case illustrates the importance of cytogenetic analysis on pleural fluid with fluorescence *in situ* hybridization (FISH) or comparative genomic hybridization (CGH) in patients with AML presenting with pleural effusion (17) to prevent misdiagnosis as drug toxicity, infection, secondary malignancy, or autoimmune phenomenon related effusions. Immunohistochemistry is also helpful (17–20). In addition to their role in diagnosis, cytogenetic characteristics may change treatment options and prognosis for subgroups of AML patients.

A recent review suggested that EML is more common than previously thought, and that routine baseline pre-treatment imaging studies with PET/CT may be warranted to help establish the true incidence of EML (21). Additionally, any patient with AML presenting with a pleural effusion should routinely undergo cytogenetic evaluation as a part of the pleural fluid analysis.

Further studies are warranted to elucidate the prognostic significance of AML pleural effusions and on the safety and efficacy of newer targeted therapies. Success has been reported with standard induction chemotherapy, stem cell transplant, and as with our case presentation, with hypomethylating agent decitabine as therapeutic options for patients with such unique presentations.

## Conclusion

Leukemic effusions are rare but the incidence may be underestimated. Patients with AML presenting with a pleural effusion should receive cytogenetic studies as a routine part of the pleural fluid analysis. Systemic chemotherapy has yielded successful results with resolution of effusions and complete remission. Further prospective studies are warranted to better characterize the incidence and outcomes of leukemic pleural and pericardial effusions from AML.

## Conflict of Interest Statement

The authors declare that the research was conducted in the absence of any commercial or financial relationships that could be construed as a potential conflict of interest.
